# Recurrent arthritis caused by *Candida parapsilosis*: a case report and literature review

**DOI:** 10.1186/s12879-019-4255-1

**Published:** 2019-07-17

**Authors:** Hang Fang, Lisi Huang, Rongkai Zhang, Denghui Xie, Hengbiao Sun, Chun Zeng, Daozhang Cai

**Affiliations:** 1 Department of Orthopaedics, The Third Affiliated Hospital of Southern Medical University, Zhongshan Ave. West, Guangzhou, 510630 People’s Republic of China; 2 Academy of Orthopaedics, Guangdong Province, 183 Zhongshan Ave. West, Guangzhou, 510630 People’s Republic of China; 3 Orthopaedic Hospital of Guangdong Province, 183 Zhongshan Ave. West, Guangzhou, 510630 People’s Republic of China; 4 Department of Clinical Laboratory, Sun Yat-Sen Memorial Hospital, Sun Yat-Sen University, 107 Yanjiang West Road, Guangzhou, 510120 People’s Republic of China; 5 Department of Clinical Laboratory, The Third Affiliated Hospital of Southern Medical University, 183 Zhongshan Ave. West, Guangzhou, 510630 People’s Republic of China

**Keywords:** Knee joint, Recurrent arthritis, *Candida parapsilosis*, Fluconazole

## Abstract

**Background:**

*Candida* arthritis is extremely rare and also represents a major challenge of diagnosis and treatment. Here we reported a rare case of recurrent arthritis caused by *Candida parapsilosis.*

**Case presentation:**

A 56-year-old Chinese male suffered from recurrent pain and swelling in his right knee after several times of “small needle-knife” acupuncture and corticosteroid injection of the joint. *Candida parapsilosis* was cultured in his synovial fluid and identified by sequencing of its Internal Transcribed Spacer (ITS) gene. Here we present the radiological characteristics, arthroscopic pictures, and synovium pathology of this patient. Also, blood test and chemical analysis of his synovial fluid were listed as well as the ITS sequence of this *Candida* species identified. The patient underwent thorough arthroscopic debridement and then set on fluconazole 400 mg daily for 12 months. His symptoms resolved and no relapse was observed on the last follow-up. Additionally, a brief but comprehensive review of *C. parapsilosis* arthritis episodes from past to now were studied.

**Conclusion:**

With the detailed clinical information reported in this case and our literature review, we hope they would add to our knowledge of *C. parapsilosis* arthritis - its clinical settings, laboratory features, radiological characteristics, arthroscopic findings and experience of management.

**Electronic supplementary material:**

The online version of this article (10.1186/s12879-019-4255-1) contains supplementary material, which is available to authorized users.

## Background

*Candida* arthritis is extremely rare and also represents a major challenge of diagnosis and treatment because the clinical manifestations, laboratory and radiologic findings are not specific and not well defined [1, 2]. Among *Candida* species, *Candida albicans* contributes the highest incidence of cases with *Candida* arthritis [3]. Whereas, the reports of *C. parapsilosis* arthritis was infrequent and limited to individual case descriptions. In this study, we reported a rare case of fungal arthritis due to *C. parapsilosis* seen at the Orthopedics Department of the Third Affiliated Hospital of Southern Medical University. Furthermore, we conducted a systematic review of *C. parapsilosis* arthritis episodes from past to now.

## Case presentation

A 56-year-old man was admitted to our hospital because of recurrent pain and impaired range of motion (ROM) of his right knee for over a year. His medical history included type 2 diabetes and hypertension which were poorly controlled. He told us his knee was mild painful and swollen at the first place about one year ago without any injury. He went to a local hospital of Traditional Chinese Medicine (TCM) and was treated with “small needle-knife acupuncture” and ozone injection into the knee joint for several times. His symptoms became better after these treatments. However, 2–3 months later, his knee pain and swelling came back and he was again treated with acupuncture and TCM plaster, as well as joint aspirations with corticosteroid injection. After these therapies his knee was painless for another 2 months before it became swollen and painful again. Approximately 5–6 times of aspirations and corticosteroid injections were given to him, but the time-period of pain-release became shorter and shorter.

On admission, he was afebrile, T 36.2 °C, BP 133/70 mmHg, P 83/min, R 16/min. His right knee joint was obviously swelling. A 3 cm × 3 cm local bump on anterolateral knee can be inspected, which was soft and painless on palpation. Joint line tenderness was present, and floating patella test was positive. His right knee has impaired ROM (100°-0–0°) and was painful when over-extension or over-flexion. Anterior drawer test, Lachman test and McMurray test were negative.

**Fig. 1:**
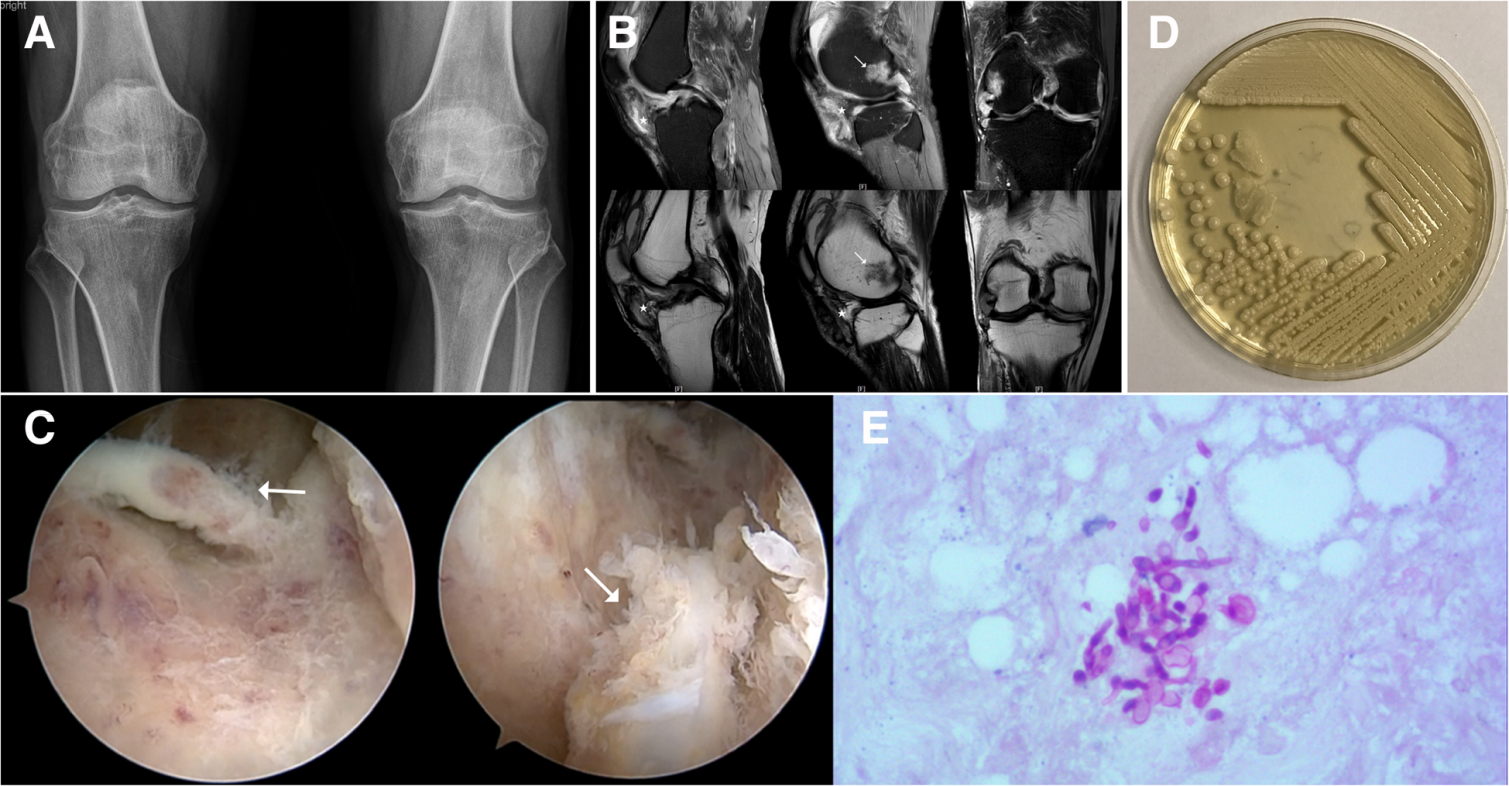
A 56-year-old man with fungal arthritis of the right knee due to *Candida parapsilosis*. **a** X-ray showed degenerative arthritis of bilateral knee. **b** MRI T2-weighted and SPAIR sequences demonstrated subchondral bone marrow edema in the lateral femoral condyle, and the presence of soft-tissue abnormalities, including capsulitis, extensive synovial hyperplasia, capsular fluid collection, and periarticular muscle edema. **c** Under the arthroscope, inflammatory synovium was observed. **d** Representative isolates of *Candida* were cultured from the synovial fluid in the Sabouraud medium. **e** PAS staining revealed the budding cells and pseudohyphae of *Candida parapsilosis* in the synovial tissue

Blood tests showed elevated erythrocyte sedimentation rate (ESR, 29 mm/h, reference range < 20 mm/h) and C-reactive protein (CRP, 18.38 mg/L, reference range < 8 mg/L), while the white blood cell (WBC, 7.3 × 10^9^/L, reference range 3.4–10.0 × 10^9^/L) count and hemoglobin (HB, 140 g/L, reference range 131–172 g/L) were normal. Radiographs of both knees exhibited the formation of osteophytes and narrowing of joint space on the medial compartments which indicated osteoarthritis (Fig. 1a). MRI T2-weighted and SPAIR sequences demonstrated subchondral bone marrow edema in the lateral femoral condyle, and the presence of soft-tissue abnormalities, including capsulitis, extensive synovial hyperplasia, capsular fluid collection, and periarticular muscle edema (Fig. 1b).

The patient then underwent thorough arthroscopic debridement and partial meniscectomy of his right knee. Inflammatory synovium was observed under the arthroscopy (Fig. 1c). Meanwhile, his thick, yellow and turbid synovial fluid was harvested. The biochemical and cytological analyses of joint fluid were as follows: Rivalta test, +++; total cell, 454 × 10^9^/L; WBC, 155 × 10^9^/L (22% mononuclear neutrophils and 78% polymorphonuclear neutrophils); protein, 37.7 g/L; lactate dehydrogenase (LDH), 1841 U/L. The Gram stain and acid-fast stain of the fluid demonstrated no bacteria or tuberculosis. Representative isolates of *Candida* were cultured in the Sabouraud dextrose agar medium (Fig. 1d). Subsequently, *Candida* species was identified by Internal Transcribed Spacer (ITS) sequencing [4] (forward primer ITS1, 5′-TCCGTAGGTGAACTTGCGG-3′; reverse primer ITS4, 5′-TCCTCCGCTTATTGATATGC-3′) and confirmed as *C. parapsilosis* by BLAST on NCBI (http://www.ncbi.nlm.nih.gov/). The sequence of ITS gene amplified from this isolate was listed in Additional file 1. Notably, the budding cells and pseudohyphae were also observed in the synovial tissue by periodic acid-schiff (PAS) staining (Fig. 1e), which further confirmed the diagnosis of Candida arthritis. Susceptibility test was performed and yielded susceptibilities to 5-flurocytosine, amphotericin B, fluconazole, itraconazole, and voriconazole.

The patient was then treated with fluconazole 400 mg daily intravenously for three weeks and then switched to orally for one year. The pain and swelling of the knee subsided gradually, and the patient had no complaints of the aforementioned symptoms 4 weeks post-surgery. On one-year follow-up, the patient remained in a clinically stable condition and the last culture of joint fluid was negative for *C. parapsilosis*.

## Discussion

In the present study, the patient manifested with a mild chronic knee arthritis characterized by recurrent pain and swelling, leading to a diagnostic delay that lasted for almost one year. Fortunately, mycologic investigation was performed in his first visit to our hospital and *C. parapsilosis* was identified in the joint fluid and synovial tissue. Previously, many lines of evidence have indicated that most patients with fungal infection are immunosuppressed with predisposing factors, including systemic disease, recent surgery, chemotherapy, long-term antibiotics use, corticosteroid therapy and insertion of central venous catheters [5, 6]. In the current case, the patient had poorly controlled blood glucose and several events of corticosteroid injection of his knee which might cause global and local impairment of immunity. Besides, TCM treatment such as “small needle-knife acupuncture” might also predispose the knee to local inoculation of pathogen if equipment was not well sterilized. Owing to these risk factors, the possibility of fungal infection should be taken into consideration.

*C. parapsilosis* is an opportunistic human pathogenic fungal species and also an uncommon cause of septic arthritis. From 1979 to 2019 there were not more than 20 cases of *C. parapsilosis* arthritis reported in the English literature. In this study, we collected 16 available cases (including one case from the current report) which the final diagnoses were made by the mycological culture and exhibited in Table 1 [7–19]. The data suggested that local pain and swelling were the most common clinical manifestations of *C. parapsilosis* arthritis, while fever and erythema occurred infrequently. Since the symptoms are always mild and unspecific, the diagnosis can often be delayed. For the patients with recurrent infection and underlying immunosuppression, clinicians should raise the suspicion of fungal infection. Moreover, our review showed that *C. parapsilosis* arthritis could occur in the knee (10/16), hip (2/16), shoulder (4/16) and wrist (1/16). Knee was the most frequently infected site, which was similar to the previous studies on Candida arthritis [3, 20], and almost all cases were monoarticular infection except one.

**Table 1:** Clinical features of the patients with *Candida parapsilosis* arthritis in the literatures

Patient No. (reference/year of publication)	Age (years) /sex	Localization of infection	Underlying conditions	Clinical manifestations	Therapy	Outcome
1 (7)/1979	64/M	Knee	Immunosuppression, intraarticular joint injections, total knee arthroplasty	Pain and swelling	Resection Arthroplasty, AMB + 5-FC for 3 months	Cured. No recurrence after 1 year of follow-up
2 (8)/1983	59/M	Shoulder	Drug abuse, hemiarthroplasty	Pain and swelling	Resection Arthroplasty, AMB followed by KZ	Cured.
3 (9)/1984	57/M	knee	End stage renal disease	Swelling	High dose of AMB for over 2 months	Remission (defer further treatment)
4 (10)/1986	70/F	Shoulder	hemiarthroplasty	Pain and swelling	Resection Arthroplasty, AMB	Cured.
5 (10)/1986	35/M	Knee	Total knee arthroplasty	Swelling	Resection Arthroplasty, 5-FC (duration NR)	NR.
6 (11)/1993	37/M	Knee	HIV infection, Hemophilia, total knee replacement, septic arthritis	Erythema and pain	Resection arthroplasty; FZ for 6 months	Cured.
7 (12)/1998	64/M	knee	Diabetes, total knee arthroplasty	swelling	Irrigation, debridement and drainage; total synovectomy; AMB for 4 months followed by FZ for 3 months	Cured. No recurrence after 2 years of follow-up
8 (13)/2001	68/F	Knee	total knee arthroplasty	Pain	Resection arthroplasty, FZ for 10 weeks, Reimplantation at 3 months	Cured.
9 (14)/2002	38/F	Knee	Diabetes, renal transplantation	Fever, swelling and tenderness	Arthroscopic irrigation, FZ + 5-FC for over 1 year	Cured. No recurrence after over 1 year of follow-up
10 (15)/2006	50/M	shoulder	Drug abuse, HIV infection	Pain with fistula	Debridement; caspofungin for 6 weeks	Cured.
11 (16)/2008	65/F	Knee and shoulder	Diabetes, secondary renal failure, renal transplantation	Pain, swelling and redness	FZ for 2 months	Cured. No recurrence after 6 months of follow-up
12 (17)/2010	66/M	Hip	Chronic kidney failure, previous Serratia marcescens prosthesis infection	Post-operative fistula	2-stage reimplantation; FZ for 6 months	Cured.
13 (17)/2010	77/F	Hip	Previous coagulase-negative Staphylococcus prosthesis infection	Fever and erythema 5 months after treatment for bacterial prosthesis infection	Resection arthroplasty; AMB + 5-FC for 2 weeks followed by FZ for 9 months	Cured.
14 (18)/2010	48/F	Knee	arthroscopic arthroplasty	Pain and swelling	AMB for 3 weeks followed by FZ for 6 months	Cured.
15 (19)/2012	60/F	Wrist	rheumatoid arthritis	Swelling	Surgical debridement and wrist arthrodesis; FZ for life	Cured.
16 (CR)	56/M	Knee	Diabetes, intraarticular joint injections	Pain and swelling	Surgical debridement; FZ for 1 year	Cured. No recurrence after 1 year of follow-up

AMB, amphotericin B; FZ, fluconazole; 5-FC, flucytosine; KZ, ketoconazole; NR, not reported; CR, current report

In addition, the cases demonstrated a wide range of underlying conditions, including surgery, diabetes mellitus, rheumatoid arthritis, renal transplantation, hemodialysis, intravenous drug addiction and HIV infection. Notably, the majority of episodes (10/16) were associated with secondary infection due to arthroplasty. Previous studies have revealed that the risk of infectious complications after joint replacement is estimated at 1–3% and fungal agents contribute 1% of prosthetic infections, among of which *C. albicans* is the most frequent species followed by *C. parapsilosis* [17, 21, 22]. It is now widely accepted that the majority of Candida infections are related to biofilm formation on the host tissue or on the surfaces of medical devices or prostheses. *C. parapsilosis* biofilms are thinner, less structured and comprised exclusively of clumped blastospores in comparison with *C. albicans*, which may be attributed to their lower pathogenicity [23]. Our review indicated that surgical treatment was an essential approach to eradicate the prosthetic infection. Out of ten patients, seven underwent resection arthroplasty, among of which one also had reimplantation. While the other two received surgical debridement, total synovectomy and two-stage reimplantation, respectively, and only one was treated successfully with antifungal agents alone. The latter patient without predisposing factors developed right knee arthritis due to *C. parapsilosis* 8 weeks after arthroscopic arthroplasty and was given amphotericin B (0.7 mg/kg/day) intravenously for 3 weeks followed by oral suppressive treatment with fluconazole (400 mg/day) for 6 months, which brought a favorable outcome without surgery [18]. However, the success rate of antifungal treatment alone is very low. Presently, the treatment of Candida prosthetic infection is still not clearly defined. In North America, the association of long-term antifungal use with two-stage exchange arthroplasty is considered as the gold standard management for prosthetic infection [24]. However, Fernando Cobo et al. pointed out that one-stage exchange arthroplasty or antifungal treatment alone was also possible to obtain a favorable outcome [25]. Additionally, all patients received antifungal therapy, with a single drug in ten cases, with two drugs in five cases and more than two in one. Among these six cases with two or more antifungal agents, only one was mentioned to have performed susceptibility test for *C. parapsilosis* isolate. As the emergence of antifungal resistance increases gradually, antifungal susceptibility testing is recognized as a useful aid in selecting the most appropriate antifungal agent, especially for treating serious forms of candidiasis [26, 27]. In our literature review, most of cases commonly began with amphotericin B or fluconazole with or without combinations, such as flucytosine. Moreover, ketoconazole was given in some cases, but we have to mention that its systemic administration is no longer recommended due to growing evidences of hepatotoxicity, endocrine dysregulation, several drug interactions, and death [28]. Furthermore, echinocandins, including caspofungin, anidulafungin, and micafungin, are promising in Candida arthritis, especially for the patients with fluconazole resistant and/or intolerant to voriconazole [15]. Recently, fluconazole is supposed to be superior to echinocandins for the treatment of *C. parapsilosis* arthritis on the basis of the decreased in vitro activity of echinocandins against *C. parapsilosis* and reports of echinocandin resistance among selected isolates [29–31]. Of special note, treatment of Candida arthritis remains a big challenge with the risk of relapse, thus a constantly long-term monitoring is still recommended.

In conclusion, our study reported the literature review of *C. parapsilosis* arthritis for the first time, which might help to add our knowledge of its clinical settings and experience of management. On the other hand, we have to admit the limitation that our review can not fully reflect the clinical characteristics of the disease since there are still a quite small number of relevant cases covered. Following the incremental use of immunosuppressive therapies together with broad-spectrum antibiotic treatment as well as the increasing number of patients implanted with joint arthroplasties, the incidence of *C. parapsilosis* arthritis has increased gradually. The timely diagnosis is of great importance to help initiate antifungal therapy promptly, prevent cartilage destruction as well as preserve joint functions.

## Additional file

Additional file 1: The sequence of ITS gene amplified from the representative isolate of *Candida*. (TXT 493 bytes)

## Data Availability

The data that support the findings of this study are available from the corresponding author (DZC) upon reasonable request.

## Abbreviations

CRP: C-reactive protein
ESR: Erythrocyte sedimentation rate
HB: Hemoglobin
ITS: Internal Transcribed Spacer
LDH: Lactate dehydrogenase
PAS: Periodic acid-schiff
ROM: Range of motion
TCM: Traditional Chinese Medicine
WBC: White blood cell
